# Electro-Tactile Stimulation Enhances Cochlear Implant Speech Recognition in Noise

**DOI:** 10.1038/s41598-017-02429-1

**Published:** 2017-05-19

**Authors:** Juan Huang, Benjamin Sheffield, Payton Lin, Fan-Gang Zeng

**Affiliations:** 1 Mind and Brain Institute, Department of Biomedical Engineering, Johns Hopkins University, Baltimore, MD USA; 2 Army Hearing Division, US Army Public Health Center, Aberdeen, MD USA; 3 National Military Audiology and Speech Pathology Center, Walter Reed National Military Medical Center, Bethesda, MD USA; 4 Center for Information Technology Innovation, Academia Sinica, Taipei, Taiwan; 5 Departments of Anatomy and Neurobiology, Biomedical Engineering, Cognitive Sciences, and Otolaryngology – Head and Neck Surgery, Center for Hearing Research, 110 Medical Science E, University of California, Irvine, California 92697-5320 USA

## Abstract

For cochlear implant users, combined electro-acoustic stimulation (EAS) significantly improves the performance. However, there are many more users who do not have any functional residual acoustic hearing at low frequencies. Because tactile sensation also operates in the same low frequencies (<500 Hz) as the acoustic hearing in EAS, we propose electro-tactile stimulation (ETS) to improve cochlear implant performance. In ten cochlear implant users, a tactile aid was applied to the index finger that converted voice fundamental frequency into tactile vibrations. Speech recognition in noise was compared for cochlear implants alone and for the bimodal ETS condition. On average, ETS improved speech reception thresholds by 2.2 dB over cochlear implants alone. Nine of the ten subjects showed a positive ETS effect ranging from 0.3 to 7.0 dB, which was similar to the amount of the previously-reported EAS benefit. The comparable results indicate similar neural mechanisms that underlie both the ETS and EAS effects. The positive results suggest that the complementary auditory and tactile modes also be used to enhance performance for normal hearing listeners and automatic speech recognition for machines.

## Introduction

Users of modern cochlear implants perform well in speech recognition tasks in quiet, but are limited in pitch-related tasks^1–3^. Electric pitch perception is limited by the electrode-to-nerve-interface, which currently does not provide access to low-frequency spiral ganglion neurons that are located in either the core of the auditory nerve bundle or the distal side of the internal auditory canal^4^. For those with residual acoustic hearing at lower frequencies, electro-acoustic stimulation (EAS) is an effective approach to access these low-frequency neurons^5^. The EAS combination of unintelligible low-frequency acoustic hearing and electric stimulation has been shown to provide a super-additive effect that improves speech recognition in noise^6–9^. However, the benefits of EAS are not readily available for those without any functional low-frequency acoustic hearing. Although penetrating electrodes have been previously proposed to directly access the low-frequency cells, mismatches between the hard electrodes and the soft tissue limits its immediate clinical application^10^. Here we consider an alternative strategy, namely, electro-tactile stimulation (ETS) that uses tactile vibrations to provide the low-frequency acoustic information.

Historically, tactile aids have competed with cochlear implants for providing auditory rehabilitation for those with profound hearing loss^11–14^. Modern advances in cochlear implants have now phased out the use of tactile aids. However there are several reasons for reconsidering tactile aids as a complementary mode to cochlear implants. First, tactile sensation is a low-frequency channel that operates in the same range (<500 Hz) as the acoustic frequencies in the EAS approach^15^. Second, tactile stimulation has been shown to convey some acoustic information that can benefit speech recognition, lipreading, and even word acquisition^16–19^. Third, it is especially interesting to note that tactile stimulation by converting voice pitch into vibration patterns improves discrimination of speech intonation contrasts^20, 21^, an approach that is similar to the demonstrated role of fundamental frequency in the EAS benefits^22, 23^.

**Figure 1:**
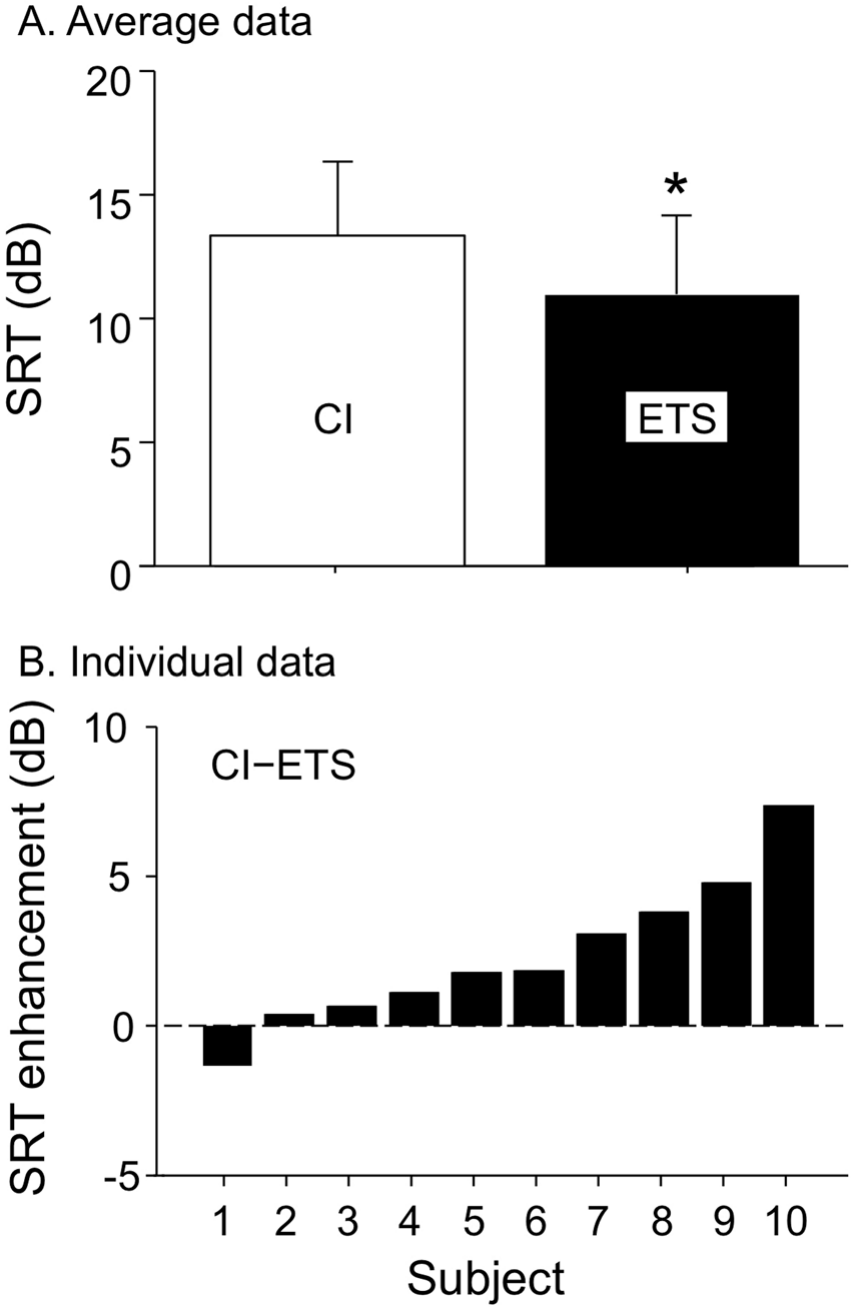
(**A**) Average speech reception threshold (SRT) between the cochlear implant only condition (CI: open bar) and the combined electro-tactile stimulation (ETS: filled bar). The error bars represent one standard deviation of the mean. The asterisk indicates a significant improvement of the ETS condition over the CI condition. (**B**) Individual enhancement in terms of the SRT difference between the CI and ETS conditions, ranked from low (Subject 1) to high (Subject 10).

Here we extracted the fundamental frequency of speech sentences and converted it into tactile vibrations that were delivered to the index finger of ten cochlear implant users. We compared speech recognition in noise with cochlear implants alone and with the additional tactile stimulation. On average (Fig. 1A), the speech reception threshold was 13.1 dB for the cochlear implant alone condition, which was significantly worse than the 10.9 dB for the bimodal ETS condition (size of the effect = 2.2 dB: paired t-test (9) = 2.00, *p* < 0.05). On an individual basis (Fig. 1B), except for Subject 1, who displayed worse performance with the additional tactile stimulation (−1.2 dB), all subjects showed improved performance from 0.3 dB (Subject 2) to 7.0 dB (Subject 10) for the bimodal ETS condition.

## Discussion

### Comparison with electro-acoustic stimulation

For EAS, the amount of potential improvement is known to depend on the quality of the low-frequency hearing. Under optimal EAS conditions simulated by normal-hearing subjects, low-frequency acoustic sounds can improve speech reception threshold by 10–15 dB^24^. A similar effect has also been observed when using only the voice fundamental frequency^23, 25^. For actual EAS users, the enhancement effect was reduced to 1–5 dB^26–29^, likely due to impairments in residual acoustic hearing^30^. The present 2.2 dB ETS effect is within the range as previously reported in actual EAS users.

### Underlying mechanisms

The similar range of improvement for both ETS and EAS suggests the involvement of similar underlying mechanisms. First, ETS and EAS both utilize the same low- frequency range (<500 Hz). Second, compared with the auditory mode, the tactile mode produces similar intensity discrimination of 1–3 dB^31^ and gap detection of 10 ms^32^ at comfort levels. However, tactile frequency discrimination is more than one order of magnitude worse (~20%) compared to the 1% or less difference limen in acoustic hearing^33, 34^. In other words, tactile stimulation should only be considered as a spectrally-impaired channel for auditory information, with a psychophysical capacity similar to the actual EAS users. Third, tactile information is known to integrate with auditory information throughout the auditory pathway from the cochlear nucleus to the auditory cortex^35, 36^. Finally, tactile stimulation affects auditory perception from sound detection and discrimination to speech recognition and even tinnitus generation^37–40^. These bimodal interactions are likely the neural basis underlying the present ETS effect.

### Design considerations for electro-tactile stimulation

In order to provide full-spectrum information for speech recognition, previous tactile aids had over-ambitious goals^41^ with designs having multiple contacts and complex stimulation patterns^42^. In contrast, the present ETS results suggest that tactile aids should be designed with different goals when integrated with cochlear implants. Due to the limited tactile capacity and the proven fundamental frequency advantage, tactile aids only need to provide low-frequency information to convey voice pitch with matched tactile capacity. For instances, in speakers, such as some females and children, with a fundamental frequency over 200 Hz, the tactile aid can transpose the fundamental frequency to a lower frequency range (e.g., <200 Hz) that is the most sensitive to touch^43^, while providing similar enhancement of cochlear implant performance as shown in previous EAS studies^25^. Alternatively, the temporal patterns of the fundamental frequency can instead be converted into spatial patterns^44^. Because vibrotactile and electrotactile modes have both shown similar perceptual capacity^45^, future studies may consider the delivery of electrotactile stimulation as an integrated tactile aid and cochlear implant option. Finally, tactile aids can be incorporated in future human and machine interface systems^46–48^.

## Methods

### Subjects

Ten cochlear implant subjects participated in this study, including 7 females, and 3 males with ages ranging from 35 to 82 years old. The subjects used either a Nucleus device (Cochlear Ltd., Sydney, Australia) or a Clarion device (Advanced Bionics Corp. Valencia, CA). They had over one year of experience with their respective devices and performed well on HINT sentences in quiet (82 ± 5% correct recognition scores). The subjects had an unaided air-conduction threshold that was greater than 80 dB HL at octave frequencies from 125 Hz to 8000 Hz. All subjects signed an informed consent approved by the University of California Irvine Institutional Review Board (IRB) and were paid for their participation in the study. The IRB approved the experimental protocol used in the present study, ensuring compliance with federal regulations, state laws, and university policies.

### Stimuli

**Figure 2:**
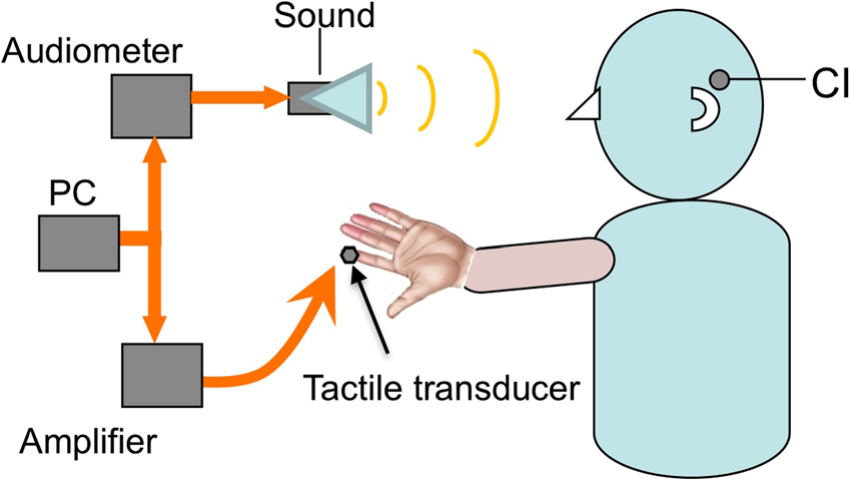
The ETS experimental setup. A personal computer (PC) controls both electric and tactile stimulation. Electric stimulation is delivered to a cochlear implant (CI) through an audiometer and speaker. Tactile stimulation is delivered to the index finger through an amplifier and a tactile transducer.

Figure 2 illustrates the experimental setup. A computer was used to control the stimulus generation, calibration, and delivery through custom Matlab programs and a 24-bit external USB sound card at a 44.1 kHz sampling rate (Creative Labs Inc., Milpitas, CA). Auditory stimulation was delivered via a GSI 61 audiometer and speaker (Grason-Stadler Inc., Eden Prairie, MN). The subjects were placed in a soundproof booth at a distance of 1 meter away from the speaker. The most comfortable level was presented on an individual basis, ranging from 65 to 75 dB SPL across subjects.

A tactile transducer (Tactaid Model VBW32, Audiological Engineering Corp., Somerville, MA) was used to deliver tactile stimulation. The tactile transducer was powered by an amplifier (Crown Audio, Elkhart, IN), and attached to the index fingertip of the non-dominant hand of the subject using electrical tape. The subjects rested their arms on a desk and were asked to place their hand palm-side up to keep the vibration intensity consistent. A 250-Hz sinusoid was used to calibrate the tactile stimulation, with the maximum output of the tactile stimulator being adjusted to 2.5 volts, or a 0 dB reference. The most comfortable level of tactile stimulation ranged between −20 dB to −10 dB relative to the maximum output across the subjects.

IEEE sentences^49^ were used as the target stimuli while speech-spectrum-shaped noise was used as the masker. Due to the limited bandwidth of tactile sensation^15^, only the fundamental frequency of the IEEE sentences was extracted and delivered to the tactile transducer. The method of fundamental frequency extraction was described previously^22, 50^. To deliver ETS, the unprocessed IEEE sentences were presented to the cochlear implants while the fundamental frequency was delivered simultaneously to the tactile transducer.

### Procedures

A one-down, one-up adaptive procedure was used to measure the speech reception threshold^51^. Speech reception threshold was defined as the signal-to-noise ratio at which the subject achieved 50% correct responses. Therefore, lower speech reception thresholds meant better performance.
